# A novel pathogenic *APC* variant identified in a Chinese pedigree with familial adenomatous polyposis

**DOI:** 10.3389/fgene.2026.1776361

**Published:** 2026-03-16

**Authors:** Chenyu Zhao, Chiyu Cai, Meng Xie, Bing Bai, Shengli Kuang, Dongxiao Li, Hui Huang

**Affiliations:** 1 Department of Gastroenterology, Henan Provincial People’s Hospital, People’s Hospital of Zhengzhou University, Zhengzhou, Henan, China; 2 Department of Hepatobiliary and Pancreatic Surgery, Henan Provincial People’s Hospital, People’s Hospital of Zhengzhou University, Zhengzhou, Henan, China; 3 Department of Oncology, Henan Provincial People’s Hospital, People’s Hospital of Zhengzhou University, Zhengzhou, Henan, China; 4 Department of Medical Genetics, Hunan Province Clinical Research Center for Genetic Birth Defects and Rare Diseases, The Second Xiangya Hospital, Central South University, Changsha, Hunan, China

**Keywords:** APC gene, colorectal cancer, familial adenomatous polyposis, microsatellite instability high, variant

## Abstract

**Background:**

Familial adenomatous polyposis (FAP) is an autosomal dominant genetic disorder characterized by the development of numerous colorectal polyps and a high predisposition to colorectal cancer, primarily caused by germline variants in the *APC* gene. This study aimed to identify and functionally validate a novel *APC* variant in a Chinese FAP pedigree.

**Methods:**

A three-generation Chinese FAP pedigree was recruited. Peripheral blood samples were collected from family members to extract genomic DNA. Whole-exome sequencing (WES) was performed to screen candidate variants, and Sanger sequencing was used for verification. SW480 cells (endogenously deficient in functional APC) were divided into three groups: empty vector group, APC-wild-type (APC-WT) group, and APC-mutant group. Western blot analysis was conducted to detect β-catenin protein expression levels, to evaluate the functional impact of the identified variant.

**Results:**

The proband’s FAP-associated colorectal cancer was identified as exhibiting microsatellite instability high (MSI-H) with a classic MLH1/PMS2 dual loss pattern. A novel germline variant *APC* c.3799dup was identified in all affected family members but was absent in unaffected individuals. Western blot analysis showed that β-catenin protein levels in the APC-WT group were significantly lower than those in the APC-Mutant group (P < 0.05) and the empty vector group (P < 0.01). This indicated that the c.3799 dup variant abolished APC’s ability to promote β-catenin degradation, leading to sustained activation of the Wnt/β-catenin pathway.

**Conclusion:**

The novel *APC* variant c.3799 dup is a pathogenic variant associated with FAP. Our findings expand the spectrum of known *APC* variants and provide functional evidence for the pathogenicity of this variant. The rare co-occurrence of FAP and MSI-H in the proband enriches the molecular phenotypic spectrum of FAP-related tumors.

## 1 Introduction

Familial adenomatous polyposis (FAP; OMIM#175100) is an autosomal dominant inherited disease characterized by the early-onset development of numerous adenomatous polyps (typically 100–1000) in the colon and rectum. It typically manifests during adolescence, with an average age of onset of 16 years (Majumder et al., 2018). It was first described in 1925, with an incidence of 3–10/100,000 worldwide (Chen et al., 2015; Wang et al., 2019). FAP is a colorectal cancer predisposition syndrome that accounts for approximately 1% of all colorectal cancers (CRCs) (Zhang et al., 2016). The molecular pathogenesis of FAP is predominantly attributed to germline variants in the adenomatous polyposis coli (*APC)* gene, a crucial tumor suppressor gene. Identified as the causative gene for FAP in 1991 (Plawski et al., 2013), *APC* encodes an APC protein that functions as an antagonist of the Wnt/β-catenin signaling pathway, which plays a pivotal role in regulating cell proliferation. In FAP patients, loss-of-function or truncated APC proteins disrupt this regulatory mechanism, leading to an abnormal accumulation of β-catenin in the cytoplasm. This dysregulation of the Wnt/β-catenin signaling cascade subsequently drives uncontrolled cell proliferation, facilitating the progression and development of colon cancer (Zhang et al., 2017). To date, the Human Gene Mutation Database (HGMD) has cataloged 2758 mutations in the *APC* gene. In this study, we investigated a family affected with FAP. A novel disease-causing variant, c.3799 dup (g.131896_131897insA; p.T1267Nfs*9), in the *APC* gene (NM_000038.6) was identified in this pedigree.

## 2 Materials and methods

### 2.1 Patients and sample collection

**FIGURE 1:**
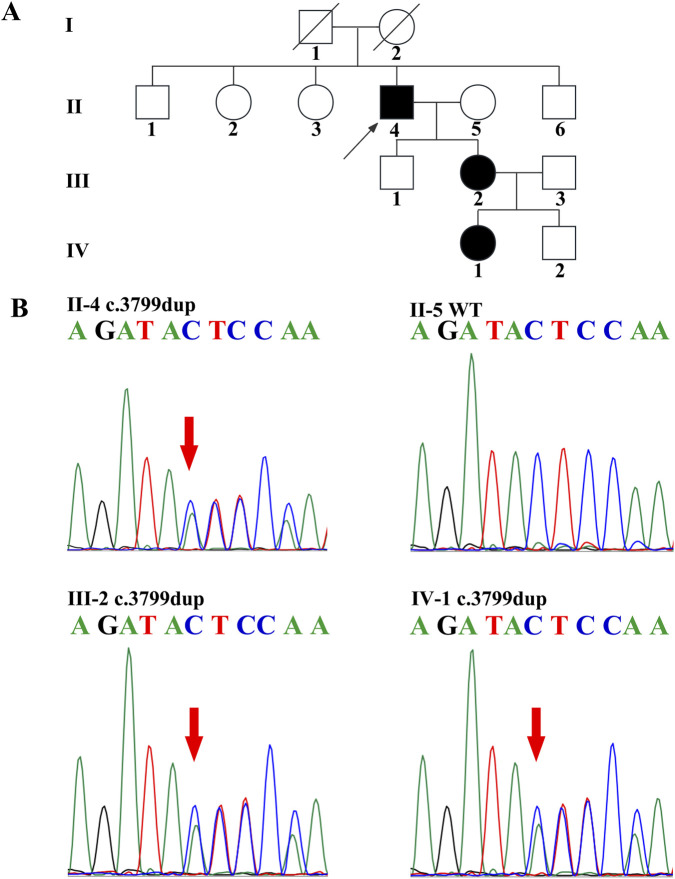
FAP pedigree and Sanger sequencing of *APC* c.3799dup variant **(A)**. The FAP pedigree. The arrow denotes the proband **(B)**. **(B)** Sanger sequencing revealed a heterozygous c.3799 dup variant (indicated by the arrow) in the proband, III-2, and IV-1, while II-5 had wild-type sequences.

A Chinese pedigree affected by FAP was obtained from Henan Provincial People’s Hospital (Figure 1A). The clinical diagnosis of FAP adhered to the following established criteria: (1) individuals presenting with more than 100 polyps or adenomas in the colorectum; or (2) those with a family history of FAP and at least 20 polyps or adenomas in the colorectum. For the purpose of genetic testing, blood samples were collected from family members II-4, II-5, III-2, and IV-1 and subsequently sent to the Second Xiangya Hospital. Unfortunately, blood samples from other family members were not available for analysis.

### 2.2 Genetic testing methods

The genetic testing methods were described in our previous study (Zhao et al., 2020; Zhao et al., 2022). We extracted genomic DNA from whole blood using a Blood gDNA Mini Kit (Beiwo, Hangzhou, China). Whole-exome sequencing (WES) was performed on III-2 at the Second Xiangya Hospital. High-throughput sequencing was conducted by using the NovaSeq 6000 sequencing system (Illumina, San Diego, United States). Basic bioinformatics analysis, including read alignment to the human reference genome (GRCh37/hg19), variant calling, and annotation, was also completed by the Second Xiangya Hospital.

The filtering steps for WES data were performed according to previously described methods (Zhao et al., 2020; Zhao et al., 2022). The pathogenicity of the remaining candidate variants was comprehensively interpreted in strict accordance with the standards and guidelines for the interpretation of sequence variants developed jointly by the American College of Medical Genetics and Genomics (ACMG) and the Association for Molecular Pathology (AMP) (Richards et al., 2015).

Validation of the candidate variant identified by WES was performed via Sanger sequencing in all available family members (II-4, II-5, III-2, and IV-1). Primer pairs flanking the candidate locus were designed using Primer3 software and synthesized by Sangon Biotech (Shanghai, China). The primer sequences used in this study were previously published (Iordache et al., 2018) and are detailed in Table 1. PCR products were sequenced by a Superyears Classic Genetic Analyzer (Superyears, Nanjing, China).

**TABLE 1:** Primer sequences used for mutation sequencing of *APC* gene.

Mutations in *APC*	Primer sequences 5'→3'
Exon 16	F: GCCACAGATATTCCTTCATCACA
	R: TGCCTGGCTGATTCTGAAGA

Abbreviations: F, forward; R, revers.

### 2.3 Plasmid construction

The pCMV-APC-3Flag-Neo vector and empty vector (pCMV-3Flag) were commercially purchased from Chuling (Wuhan, China). The APC-Mutant (c.3799_3800insA) vector was constructed using a site-directed mutagenesis kit (Vazyme, Nanjing, China). Recombinant vectors were transformed into *E. coli* DH5α competent cells, and positive clones were verified by Sanger sequencing to confirm the presence of the target mutation without off-target alterations.

### 2.4 Cell culture and transfection

SW480 cells (Procell, Wuhan, China) were cultured in DMEM medium supplemented with 10% fetal bovine serum (FBS) and 1% penicillin-streptomycin at 37 °C in a 5% CO_2_ atmosphere. Cells were divided into three groups: the empty vector group (transfected with pCMV-3*Flag), the APC-wild-type (APC-WT) group, and the APC-Mutant group. For transient transfection, cells were transfected using Lipofectamine 3000 (Thermo Fisher Scientific, Waltham, United States) following the manufacturer’s instructions.

### 2.5 Western blot analysis

Total proteins were extracted with RIPA lysis buffer (containing protease inhibitors) and quantified by BCA assay. Equal protein (30 μg/lane) was separated by 10% SDS-PAGE, transferred to PVDF membranes, blocked with 5% non-fat milk (TBST, 1 h RT), and incubated with primary antibodies against β-catenin (1:1000 dilution, CST, #8480) and β-actin (1:20000 dilution, Proteintech, 66009-1-Ig) at 4 °C overnight. After washing, the membranes were incubated with horseradish peroxidase (HRP)-conjugated secondary antibody (1:10000 dilution, Servicebio, GB23301 and GB23303) for 1 h at RT. Bands were visualized by ECL, quantified via ImageJ (β-actin as internal control), and experiments were repeated 3 times.

### 2.6 Statistical analysis

Data were analyzed with GraphPad Prism 9.0 (San Diego, United States). All data were presented as the mean ± standard error of the mean (SEM). One-way analysis of variance (ANOVA) followed by Tukey’s multiple comparison test was used to compare the differences. The criterion for statistical significance was p < 0.05.

## 3 Results

### 3.1 Clinical evaluation

**FIGURE 2:**
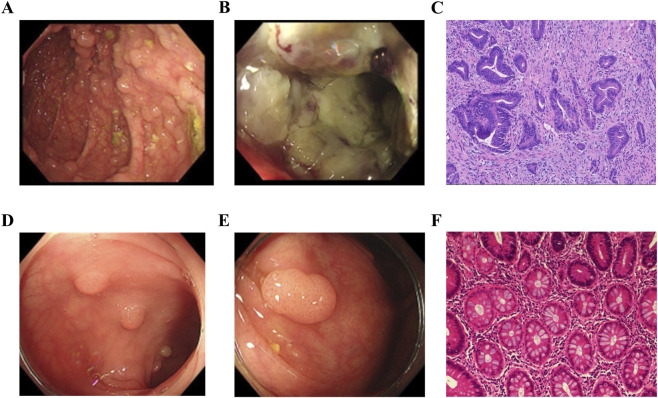
Clinical and histopathological manifestations of the colon in FAP family members. **(A)** Multiple polyps in the transverse colon (II-4). **(B)** Adenocarcinoma in the ascending colon (II-4). **(C)** Histopathological results revealed adenocarcinoma (II-4). **(D)** Multiple polyps in the transverse colon (III-2). **(E)** Multiple polyps in the ascending colon (III-2). **(F)** Histopathological results revealed tubular adenoma (III-2).

A 45-year-old Chinese male patient (II-4, Figure 1A), born to nonconsanguineous parents, presented to Henan Provincial People’s Hospital. At the age of 45 years, he was clinically diagnosed with FAP and concurrent colorectal cancer. He reported intermittent abdominal pain for more than 2 months, accompanied by a 4 kg weight loss. Chest, abdomen, and pelvic CT revealed uneven thickening and heterogeneous enhancement in the ascending colon and hepatic flexure, along with a space-occupying lesion in the rectum, consistent with malignant tumors. Colonoscopy confirmed colorectal cancer and endoscopic features typical of FAP. Pathological examination confirmed colonic adenocarcinoma with microsatellite instability high (MSI-H) (Figures 2A–C; Table 2). Immunohistochemical (IHC) staining for mismatch repair (MMR) proteins showed negative expression of MLH1 and PMS2, alongside positive expression of MSH2 and MSH6. MSI was examined by polymerase chain reaction (PCR) and capillary electrophoresis. The tumor was clinically staged as stage IV. Given the MSI-H subtype, he received five cycles of tislelizumab (a PD-1 inhibitor) treatment, and subsequent follow-up CT showed the disease remained stable.

**TABLE 2:** Clinical and genetic characteristics of participants in the family

Family ID	SEX	Age (years)	Clinical symptoms	No. of colorectal adenomas or polyps	Mutation in *APC*
II-4	M	45	FAP, colon cancer	>100	c.3799_3800insA
II-5	F	44	N	NA	WT
III-2	F	23	FAP	>100	c.3799_3800insA
IV-1	F	4	N (to date)	NA	c.3799_3800insA

M, male; F, female; N, normal; NA, not available; WT, wild type.

Based on clinical manifestations, family history, colonoscopic and pathological findings, his 23-year-old daughter (III-2) was also diagnosed with FAP (Figures 2D–F; Table 2). At the time of diagnosis, hundreds of flat and hemispherical polyps measuring 3 mm to 1 cm in diameter were extensively distributed throughout her colon and rectum. We recommended total proctocolectomy with ileal pouch-anal anastomosis (IPAA) as the standard surgical intervention. However, the patient declined this operation due to her young age. Instead, she underwent endoscopic polypectomy, and more than 80 relatively large polyps were removed by snare resection. She was advised to undergo surveillance colonoscopy at 6-month intervals, with a plan to perform total proctocolectomy and IPAA if polyp progression or dysplasia develops. In addition, IV-1, at only 4 years of age, has not yet undergone colonoscopy or CT examination. II-5 tested negative on genetic analysis and did not receive further clinical workup.

### 3.2 Genetic analyses

The list of variants detected by WES is provided in the supplementary material. The c.3799 dup variant in *APC* was interpreted as a pathogenic variant. II-4, III-2, and IV-1 carried a heterozygous c.3799 dup variant in *APC*, which was not present in II-5 (Figure 1B; Table 2).

### 3.3 Effect of mutant APC on β-catenin protein level in Wnt/β-catenin pathway

**FIGURE 3:**
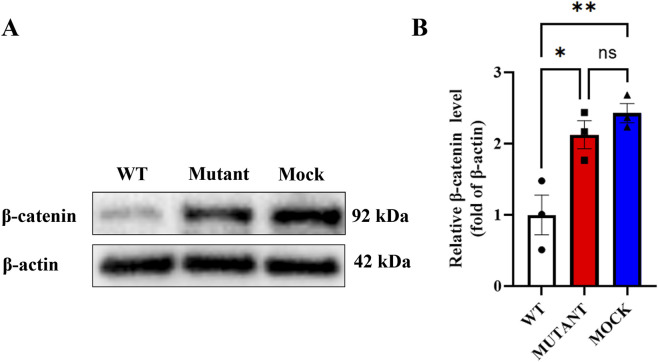
Effect of wild-type and mutant APC on β-catenin protein expression in SW480 cells. **(A)** Representative Western blot images showing β-catenin protein levels in the APC-WT group, APC-Mutant (c.3799 dup) group, and empty vector (Mock) group. β-actin was used as the internal reference to normalize protein loading. **(B)** Quantitative analysis of β-catenin protein expression. Data are presented as the mean ± standard error of the mean (SEM) from three independent biological replicates. Statistical significance was determined by one-way analysis of variance followed by Tukey’s multiple comparison test. *P < 0.05, P < 0.01 vs. the APC-WT group; ns: no significant difference between the empty vector group and APC-Mutant group.

Western blot analysis revealed significant differences in β-catenin protein expression among the three groups (Figure 3A). Gray value quantification demonstrated that compared with the APC-WT group, β-catenin levels were significantly increased in both the empty vector group (P < 0.05) and the APC-Mutant group (P < 0.05; Figure 3B). Specifically, the APC-Mutant group maintained a high β-catenin expression state, and its expression level showed no significant difference from that of the empty vector group (P > 0.05).

## 4 Discussion

FAPs are clinically classified into two distinct subtypes: classic FAP (CFAP) and attenuated FAP (AFAP). CFAP is characterized by the presence of more than 100 colorectal adenomatous polyps, whereas AFAP is defined by a lower polyp burden, typically ranging from 10 to 100 lesions. Compared with AFAP, CFAP has an earlier age of onset for both polyposis and CRC. A notable difference between these subtypes lies in the age of disease onset. Patients with CFAP generally experience the development of polyposis and colorectal cancer (CRC) at an earlier stage than those with AFAP do. In the absence of timely intervention, all FAP patients are at a near-certain risk of developing CRC. The mean age of CRC diagnosis for CFAP and AFAP patients is approximately 40 years and 55 years, respectively (Novelli, 2015; Ma et al., 2018). Beyond its colorectal manifestations, FAP is also associated with a diverse range of extracolonic symptoms. These include desmoid tumors, upper gastrointestinal polyps, lipomas, osteomas, dental anomalies, and epidermoid cysts, among others (Dinarvand et al., 2019). This multisystem involvement highlights the complex nature of FAP and underscores the importance of comprehensive clinical management for affected individuals.

**FIGURE 4:**
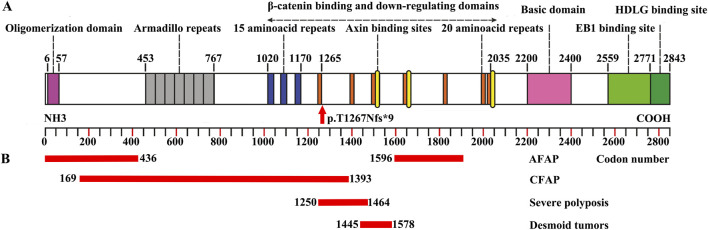
APC protein structure, variant location, and genotype–phenotype correlations. **(A)** Schematic diagram of the APC protein structure with its functional domains. The location of the variant (c.3799 dup) is indicated. **(B)** Genotype–phenotype correlations of the *APC* gene. This figure was modified from Pouya et al. (2018).

The *APC* gene, a pivotal tumor suppressor gene, is precisely mapped to the chromosomal region 5q21-q22. Comprising 16 exons, this gene encodes the APC protein, a polypeptide chain consisting of 2843 amino acid residues. The APC protein is a highly sophisticated multidomain molecule equipped with an array of binding sites that enable it to interact with numerous other proteins (Figure 4A). It can regulate β-catenin protein levels. Pathogenic variants in *APC* lead to the accumulation of β-catenin protein in the cytoplasm. β-catenin subsequently activates other transcription factors, including Tcf, thereby causing abnormal activation of the Wnt signaling pathway. It can lead to uncontrolled cell proliferation and the progression and development of colorectal cancer (Zhang et al., 2016). The c.3799 dup (p.T1267Nfs*9) variant in *APC* is a frameshift mutation that introduces a premature termination codon (PTC) 9 amino acids after the frameshift at position 1267 (Figure 4A). As highlighted, the PTC-driven truncation specifically eliminates the C-terminal region of the APC protein—this region harbors non-redundant functional domains (β-catenin-binding, Axin-binding domains) essential for Wnt pathway suppression, which aligns with our prior inference of loss-of-function (LOF).

The Western blot analysis of β-catenin expression provides critical functional evidence supporting the pathogenicity of the APC c.3799 dup variant and elucidating the underlying mechanism of FAP. Our results demonstrated that β-catenin protein levels were significantly lower in the APC-WT group compared to both the empty vector and APC-Mutant groups, with no statistical difference between the latter two groups. These findings indicate that APC-WT effectively promotes β-catenin degradation, whereas the empty vector and mutant APC both lose the ability to regulate β-catenin, resulting in sustained activation of the Wnt/β-catenin pathway. The variant was evaluated using two widely recognized bioinformatics *in silico* tools, MutationTaster (Schwarz et al., 2010) and CADD (Schubach et al., 2024), both of which consistently predicted pathogenic effects.

The c.3799 dup is a frameshift variant. This variant leads to the loss of function of the APC protein, which is a well-established pathogenesis underlying FAP, fulfilling the very strong pathogenic evidence (PVS1) criterion of the ACMG guidelines. Additionally, functional studies in our experiments demonstrated that the mutant APC loses its ability to regulate β-catenin degradation, resulting in sustained activation of the Wnt/β-catenin pathway, which aligns with the pathogenic strong evidence (PS3) criterion. This variant is absent from the control datasets of the EXAC and 1000G databases, satisfying the pathogenic moderate evidence 2 (PM2) criterion. Moreover, genetic segregation analysis revealed that the c.3799 dup variant cosegregates with the FAP phenotypes in both the proband and the affected family member but is not detected in the unaffected family member, thereby meeting the pathogenic supporting evidence 1 (PP1) criterion. The prediction software programs—MutationTaster and CADD—further indicate that the c.3799 dup variant exerts a deleterious effect, which supports the application of the Pathogenic Supporting Evidence 3 (PP3) criterion. Per ACMG’s variant classification framework, the combination of 1 very strong criterion (PVS1) + 1 strong evidence (PS3) + 1 moderate criterion (PM2) + 2 supporting criteria (PP1 and PP3) meets the threshold for pathogenic classification. Therefore, in accordance with ACMG guidelines, the variant is classified as a pathogenic variant. By searching previously published studies and public databases—including HGMD, ClinVar, gnomAD, 1000G, and ExAC—we confirmed that this is a previously unreported novel variant. An insertion mutation at this position has been previously reported, but the inserted base differs (Friedl et al., 2001).

In FAP, there is a direct correlation between genotype and phenotype (Figure 4B) (Pouya et al., 2018). Variants in the *APC* gene associated with AFAP have mostly been identified before codon 157, in exon 9 or after codon 1595 (Bodmer, 1999; Nieuwenhuis and Vasen, 2007). This genotype‒phenotype correlation is highly important for making the most suitable treatment decisions. The patients in this study carried the p.T1267Nfs*9 variant, which suggested severe polyposis.

The concurrent MSI-H status in the proband’s FAP-associated colorectal cancer is an unusual and noteworthy finding, as FAP tumors are typically microsatellite stable (MSS). We thus investigated the potential underlying mechanisms. The proband’s MMR IHC showed a classic MLH1/PMS2 dual deletion pattern, which is most commonly associated with sporadic colorectal cancer caused by MLH1 promoter hypermethylation. In FAP patients, long-term intestinal mucosal dysplasia and adenoma-carcinoma sequence may induce sporadic epigenetic alterations of *MMR* genes in tumor cells, leading to the loss of MLH1 expression and subsequent MSI-H phenotype. Besides, somatic mutations of the *MLH1* or *PMS2* gene in the proband’s colorectal cancer cells may also lead to MSI-H phenotype. And the long-term genomic instability caused by FAP-related *APC* gene mutation may further promote the occurrence of somatic *MMR* gene mutations in tumor cells, resulting in the coexistence of FAP and MSI-H phenotypes. Notably, combined with clinical, pedigree and molecular detection findings, germline *MMR* gene mutations and concurrent Lynch syndrome were excluded in this pedigree.

This study shows a rare case of FAP-associated colorectal cancer with a concurrent MSI-H phenotype, which enriches the molecular phenotypic spectrum of FAP-related tumors (traditionally considered MSS). The identification of this rare phenotype has important clinical implications: (1) For FAP patients diagnosed with colorectal cancer, MSI detection and MMR protein IHC should be routinely performed to guide individualized chemotherapy; (2) The co-occurrence of FAP and MSI-H suggests that the molecular mechanism underlying FAP-related tumorigenesis is more complex than previously thought, and genomic instability caused by *APC* mutation may cooperate with *MMR* gene abnormalities to promote tumor progression.

A key limitation of this study is the absence of targeted tumor sequencing and MLH1 promoter methylation detection for the proband’s tumor tissue due to practical clinical constraints, which prevented us from definitively determining the molecular cause of the MSI-H phenotype in this FAP-associated colorectal cancer. We were thus unable to conclusively verify whether the MSI-H status resulted from sporadic epigenetic alterations or somatic *MMR* gene mutations. Future studies with extended molecular testing and long-term family follow-up are needed to clarify the exact mechanism underlying this rare co-occurrence of FAP and MSI-H.

In conclusion, we described a family with FAP harboring a novel and pathogenic *APC* variant, and we also identified a concurrent MSI-H phenotype in the FAP-associated colorectal cancer case from this family. These results are helpful for conducting genetic diagnosis and counseling for FAP families, enrich the spectrum of *APC* variants, and further expand our understanding of the rare co-occurrence of FAP and MSI-H, which provides new insights into the molecular heterogeneity and tumorigenic mechanisms of FAP-related tumors.

## Data Availability

The original contributions presented in this study are publicly available. This data can be found at the NCBI SRA database with the accession number PRJNA1344596.
